# Team cohesion and mental resilience among vocational college students in ultimate frisbee courses: the parallel mediating roles of basic psychological need satisfaction in exercise and intrinsic motivation

**DOI:** 10.3389/fpsyg.2026.1752087

**Published:** 2026-03-20

**Authors:** Guangzhong Chen, Yanan Sun, Siti Zobidah Omar

**Affiliations:** Faculty of Social Sciences and Liberal Arts, UCSI University, Kuala Lumpur, Malaysia

**Keywords:** basic psychological need satisfaction in exercise, intrinsic motivation, mental resilience, team cohesion, ultimate frisbee, vocational college students

## Abstract

University students are increasingly exposed to academic, social and career-related stressors, and identifying mechanisms that promote their capacity to adapt to adversity has become a key priority in higher education. Ultimate frisbee, a cooperative team sport that emphasises fair play and communication, may provide a favourable context for developing mental resilience. This cross-sectional study examined how perceived team cohesion is related to mental resilience among vocational college students enrolled in ultimate frisbee courses, and whether basic psychological need satisfaction in exercise and intrinsic motivation operate as parallel mediators. A total of 390 students from five vocational colleges in Guangxi, China, completed validated Chinese versions of the Group Environment Questionnaire (team cohesion), the Basic Psychological Needs in Exercise Scale, the intrinsic motivation subscale of the BREQ-3, and the 10-item Connor–Davidson Resilience Scale. Partial least squares structural equation modelling (SmartPLS 4.0) was used to estimate a parallel mediation model. The model explained 21.8% of the variance in basic psychological need satisfaction, 43.9% in intrinsic motivation and 46.4% in mental resilience. Team cohesion showed a significant direct positive effect on mental resilience (*β* = 0.279) and was positively associated with basic psychological need satisfaction (*β* = 0.467) and intrinsic motivation (*β* = 0.663). Both mediators, in turn, positively predicted mental resilience (*β* = 0.227 and *β* = 0.320, respectively). Bootstrap analyses indicated that basic psychological need satisfaction and intrinsic motivation each exerted significant partial mediating effects between team cohesion and mental resilience. These findings highlight the psychological benefits of highly cohesive ultimate frisbee course(s) in vocational colleges and suggest that fostering supportive, need-satisfying team climates may be an effective pathway to enhancing students’ mental resilience.

## 1 Introduction

### 1.1 Background and research questions

University students worldwide are facing increasing mental health challenges (Bantjes et al., 2022; Pillay, 2022). Large-scale surveys and systematic reviews have reported high and, in some contexts, rising rates of depression, anxiety and stress-related symptoms in this population, often exceeding those observed in the general public (Varma et al., 2021; Sanni, 2025). Beyond the presence or absence of psychiatric disorders, researchers have highlighted mental resilience—an individual’s capacity to adapt, recover and grow when confronted with adversity—as a crucial psychological resource that supports academic functioning, social adjustment and overall well-being (Afita and Nuranasmita, 2023; Hu et al., 2025). Understanding how everyday educational experiences can foster mental resilience has therefore become an important agenda for higher education and student affairs.

Within the Chinese higher education system, students in vocational colleges constitute a large yet relatively vulnerable subgroup. On the one hand, vocational colleges are strongly employment-oriented, and students are required to engage early in skills training, practical assessments, internships and job-seeking activities (Pan et al., 2023; Sultana, D., 2022). On the other hand, persistent social stereotypes and hierarchical views of vocational education may undermine students’ self-esteem, self-identity and perceived social status (Bao et al., 2023; Yun et al., 2025). Existing studies suggest that some vocational college students experience higher levels of psychological distress and lower perceived life satisfaction compared with their counterparts in academic-track universities (Xu et al., 2024; Ulmanen et al., 2024). How to make better use of resources that already have wide coverage in the curriculum—especially physical education courses—to strengthen vocational students’ mental resilience and psychological health has thus become a pressing practical question (Li, 2024; Huang, 2024). In vocational colleges, students’ schedules are often organised around skills training and practical assessments, which makes time use more fragmented. PE electives therefore represent one of the few routine curriculum resources that can reach students consistently while providing structured opportunities for peer interaction and support (Yan et al., 2024).

Physical activity is widely recognised as a protective factor for university students’ mental health (Herbert, 2022; Grasdalsmoen et al., 2020). Empirical evidence indicates that regular participation in physical exercise can reduce depressive and anxiety symptoms, enhance positive affect and self-efficacy, and is positively associated with mental resilience (Kim et al., 2023; Liu et al., 2024). Team sports, compared with individual activities, provide additional opportunities for cooperation, communication and social support, which may be particularly beneficial for students’ social–emotional development (Wang et al., 2024). Ultimate frisbee is a relatively new team sport in Chinese universities that emphasises cooperation, self-officiating and “Spirit of the Game” (Xu, 2025; Fozzard, 2022). Its low technical entry threshold, high degree of interaction and emphasis on respect and fairness may offer a particularly fertile context for promoting students’ psychological resources, including resilience (Amoroso et al., 2021a). Self-officiating and the “Spirit of the Game” require students to communicate and jointly uphold rules and fairness (Brooks, 2013). In PE classes, ultimate frisbee is often taught through small-group drills and game-like activities that involve frequent coordination and role rotation (Breed and Spittle, 2021). This interaction structure is likely to strengthen peer connection and makes the course a suitable context for observing variation in team cohesion (Zamecnik et al., 2024). However, empirical research on the psychological effects of ultimate frisbee courses is still scarce, and quantitative evidence focusing on vocational college students is almost non-existent. In this PE-class context, we use in-class team interaction to frame the research questions on team cohesion and mental resilience (Leo et al., 2023).

In team sport settings, team cohesion is considered a central group-level construct linking team structure with individual psychological outcomes (Lopez-Gajardo et al., 2023; McLaren and Spink, 2020). Cohesive teams are characterised by clear and shared goals, strong interpersonal bonds and a high sense of belonging among members (Gu et al., 2022). Previous studies in various sports have shown that higher team cohesion is associated with more positive affect, stronger social support, higher satisfaction and better performance among athletes (McEwan, 2020; González-García et al., 2022). It is reasonable to expect that, in regular physical education courses, students who perceive their frisbee team as cohesive may feel more supported and motivated and, consequently, more resilient when facing training difficulties, performance setbacks or academic stress. Yet, direct evidence on the relationship between team cohesion and mental resilience in non-elite, classroom-based team sport contexts remains limited, especially in vocational colleges and ultimate frisbee courses. By contrast, within the limited literature on ultimate frisbee, quantitative evidence explicitly situated in university PE classes remains scarce (e.g., Xu, 2025). This highlights the need to add classroom-based evidence on how team process variables (team cohesion) relate to mental resilience in routine PE settings.

At the same time, the psychological mechanisms through which team cohesion might enhance mental resilience are not fully understood. Drawing on Self-Determination Theory, this study focuses on two key constructs: basic psychological need satisfaction in exercise (autonomy, competence and relatedness) and intrinsic motivation. Supportive, cohesive team environments are likely to provide students with greater opportunities to experience choice and volition in participation, a sense of effectiveness in mastering frisbee skills, and meaningful connections with teammates (Amoroso et al., 2021b). These experiences can satisfy basic psychological needs in the exercise context and, in turn, foster intrinsic motivation to engage in the course for interest and personal growth rather than external pressure (Standage and Ryan, 2020). Both basic psychological need satisfaction in exercise and intrinsic motivation have been positively linked to adaptive outcomes such as persistence, enjoyment and psychological well-being (Leisterer and Gramlich, 2021). However, few studies have integrated these constructs into a single model to examine whether they jointly mediate the link between team cohesion and mental resilience in everyday physical education settings.

Against this backdrop, the present study focuses on ultimate frisbee courses in vocational colleges in Guangxi, China, and seeks to clarify the mechanism linking team cohesion and students’ mental resilience. Their course participation is often less stable than that of traditional university students. In this context, PE classes that can sustain peer support and team interaction may matter for students’ psychological adaptation and resilience (Li et al., 2025). Therefore, we examine how team cohesion relates to mental resilience, and whether basic psychological need satisfaction and intrinsic motivation provide explanatory pathways. Although the study is situated in Chinese vocational colleges and uses ultimate frisbee as the course context, it tests a transferable classroom mechanism: whether team cohesion relates to mental resilience via basic psychological need satisfaction and intrinsic motivation (Zhang, 2024). This mechanism is relevant to other team-based PE classes or high-interaction physical education settings. Ultimate frisbee is a highly cooperative team activity with dense in-class interaction (Xu, 2025). Vocational college students typically follow practice-oriented schedules, and stable participation and peer bonding can be more challenging. In authentic PE classes, this context allows a clearer examination of whether team cohesion shows a stable association with mental resilience. Specifically, we address the following research questions:

*RQ1*: In vocational college ultimate frisbee courses, does perceived team cohesion significantly and positively predict students’ mental resilience?

*RQ2*: Do basic psychological need satisfaction in exercise and intrinsic motivation function as parallel mediators in the relationship between team cohesion and mental resilience?

RQ1 is addressed by testing H1, H3 and H6; RQ2 is addressed by testing H2, H4, H5 and H7. By answering these questions, this study aims to provide empirical evidence on how team processes and motivational mechanisms operate within a widely accessible physical education context to support the mental resilience of vocational college students.

### 1.2 Literature review and theoretical model

#### 1.2.1 Team cohesion and mental resilience

In sport and exercise psychology, team cohesion is one of the most widely studied group-level constructs. It is commonly defined as the degree to which team members are attracted to their group and are committed to achieving shared objectives while maintaining group unity (Ifeanyi et al., 2024; Berengüí et al., 2022). Empirical work has shown that higher levels of cohesion are associated with clearer collective goals, stronger interpersonal bonds and a greater willingness among members to remain with the team (Lakhmani et al., 2022; Mach et al., 2022). In adolescent and university samples, team cohesion has been linked to higher self-efficacy and a stronger sense of belonging, which in turn support exercise adherence and the maintenance of health-promoting behaviours (Zou et al., 2023).

Mental resilience focuses on how individuals adapt and recover when they encounter stress, failure or adversity (Ungar and Theron, 2020; Illouz, 2020). Measures such as the Connor–Davidson Resilience Scale conceptualise resilience as a multidimensional capacity that encompasses persistence, emotion regulation and positive adaptation (Velickovic et al., 2020). Studies conducted in competitive sport contexts suggest that athletes embedded in cohesive teams tend to report higher resilience and are more likely to maintain effort and engagement when facing setbacks (Sarkar and Page, 2022). Team cohesion has been found to correlate positively with indicators of mental toughness and to influence resilience indirectly through psychological mechanisms such as passion, goal commitment and social support (Gu et al., 2022).

Translating these insights to everyday physical education settings, it is plausible that students who perceive their class team as cohesive—sharing clear goals, enjoying harmonious relationships and being willing to remain in the team—will experience more support and confidence during practice and competition (Thornton et al., 2020; Kim et al., 2021). These experiences may help them cope more effectively with training challenges, performance fluctuations and wider academic stressors, thereby fostering higher mental resilience. However, quantitative evidence on this association in non-elite, classroom-based team sports, particularly in vocational colleges and ultimate frisbee courses, is still scarce. Based on the existing literature, the present study proposes that perceived team cohesion in vocational college frisbee classes will be positively related to students’ mental resilience and will exert a significant direct effect on resilience.

#### 1.2.2 Basic psychological need satisfaction in exercise and mental resilience

Self-Determination Theory (SDT) posits that autonomy, competence and relatedness are three universal basic psychological needs (Vansteenkiste et al., 2020; Olafsen et al., 2025). When these needs are satisfied in a given context, individuals are more likely to internalise values, display self-determined motivation and experience psychological well-being (Blechman et al., 2024). In the exercise domain, the Basic Psychological Needs in Exercise Scale (BPNS-Exercise) has been widely used to assess the satisfaction of these needs during physical activity. Validation studies in different countries and populations have shown that the scale has sound structural validity and reliability, and that higher need satisfaction is associated with more self-determined forms of motivation, greater persistence in exercise and better mental health outcomes.

Research with university students and athletes has indicated that when they feel they have some choice in activities (autonomy), believe they can successfully complete tasks (competence) and experience positive, supportive relationships with peers (relatedness), they are more likely to report enjoyment, engagement and psychological flourishing in sport and physical education contexts (Howard et al., 2025). In contrast, when these needs are thwarted, students may display amotivation, disengagement and negative affect.

Within vocational college frisbee courses, a cohesive team climate may help satisfy these needs. Teachers and student leaders can negotiate practice rules, provide graduated technical challenges and foster mutual support among team members. Under such conditions, students may feel more autonomous in how they participate, more competent in mastering frisbee skills and more connected with classmates, leading to higher basic psychological need satisfaction in exercise (Manninen and Campbell, 2022). This, in turn, is expected to contribute to their capacity to cope with academic and life stressors and to recover from difficulties, thereby supporting mental resilience (Fullerton et al., 2021).

Nevertheless, empirical studies that explicitly incorporate “basic psychological need satisfaction in exercise” and “mental resilience” into a single model remain limited, particularly in ordinary physical education classes and vocational college settings. Drawing on SDT and the above evidence, the present study assumes that basic psychological need satisfaction in exercise not only promotes mental resilience but also acts as a mediator between team cohesion and resilience.

#### 1.2.3 Intrinsic motivation and mental resilience

Along the motivation continuum proposed by SDT, intrinsic motivation represents the most self-determined form of motivation (Schüler et al., 2023). It refers to engaging in an activity for inherent interest, enjoyment or personal value, rather than for external rewards or pressures (Malone and Lepper, 2021; Suman, 2023). Students with high intrinsic motivation tend to persist in activities, display greater creativity and report more positive affect (Zhang et al., 2021; Amaro et al., 2023). In physical education and sport settings, intrinsic motivation has been linked to sustained participation, higher perceived competence and greater psychological well-being (Kruse et al., 2024; Méndez-Giménez et al., 2022). In contrast, motivation driven mainly by external demands or contingencies is more likely to be associated with boredom, burnout and dropout.

Recent work has begun to explore the association between exercise motivation and mental resilience. Findings from athlete samples suggest that individuals with stronger intrinsic motivation are better able to maintain effort, regulate emotions and reinterpret setbacks as opportunities for growth, resulting in higher resilience scores (Zuo and Bai, 2025). When students perceive an activity as interesting and meaningful, they are more willing to invest cognitive and emotional resources, which may help them face challenges with a more adaptive mindset (Bhardwaj et al., 2025).

In the context of ultimate frisbee courses, students who take part because they enjoy the sport, value the experience of cooperation and see the class as personally meaningful are likely to develop stronger intrinsic motivation. A cohesive team environment, characterised by frequent positive interactions, shared accomplishments and emotional support, can enhance students’ interest and engagement in the course (Chaudhry et al., 2024). Thus, team cohesion may not only have a direct effect on mental resilience but may also operate indirectly by strengthening students’ intrinsic motivation toward the frisbee class (Xu, 2025).

#### 1.2.4 Parallel mediating roles of BPNS-exercise and intrinsic motivation

SDT suggests that supportive social environments influence outcomes through at least two closely related yet conceptually distinct pathways: need satisfaction and high-quality motivation (Howard et al., 2021). In physical education classes, a cohesive and supportive team climate may simultaneously provide conditions that satisfy students’ basic psychological needs (e.g., offering choices, providing optimal challenges and nurturing warm peer relationships) and cultivate more self-determined forms of motivation (e.g., interest, enjoyment and personal endorsement of the activity) (Leo et al., 2022). These two mechanisms are correlated but not identical. Need satisfaction reflects the extent to which the environment fulfils fundamental psychological requirements, whereas intrinsic motivation captures the degree to which behaviour is driven by inherent interest and self-congruence.

Previous studies often focus on only one of these processes when explaining the impact of physical activity on psychological outcomes, such as modelling “need satisfaction → well-being” or “intrinsic motivation → exercise adherence”. Fewer studies have examined whether both need satisfaction and intrinsic motivation operate in parallel to transmit the effects of a given social context—such as team cohesion—on mental resilience or related indicators. In vocational college frisbee classes, cohesive teams may provide multiple sources of support: they can create opportunities for autonomy and competence, thereby raising basic psychological need satisfaction, and they can generate shared enjoyment, co-creation experiences and positive emotions, thereby strengthening intrinsic motivation (Amoroso et al., 2021b). Both pathways are theoretically capable of enhancing students’ ability to maintain adaptive functioning under stress.

In light of this, the present study does not assume a single chain mediation (e.g., “need satisfaction → intrinsic motivation → resilience”). Instead, it proposes a parallel mediation structure, in which basic psychological need satisfaction in exercise and intrinsic motivation each serve as separate mediators between team cohesion and mental resilience. Comparing the magnitude and significance of these two indirect paths can provide a more comprehensive picture of how SDT-related mechanisms operate in vocational ultimate frisbee courses and offer more targeted implications for instructional design.

#### 1.2.5 Theoretical model and hypotheses

On the basis of the above literature review and theoretical reasoning, this study constructs a theoretical model with four latent variables: team cohesion (independent variable), basic psychological need satisfaction in exercise and intrinsic motivation (parallel mediators), and mental resilience (dependent variable). The model assumes that team cohesion has a direct positive effect on mental resilience and indirect effects through the two mediators. In cohesive ultimate frisbee course(s), students are expected to experience higher levels of need satisfaction and intrinsic motivation, which in turn will enhance their mental resilience (Wolf et al., 2025).

**Figure 1:**
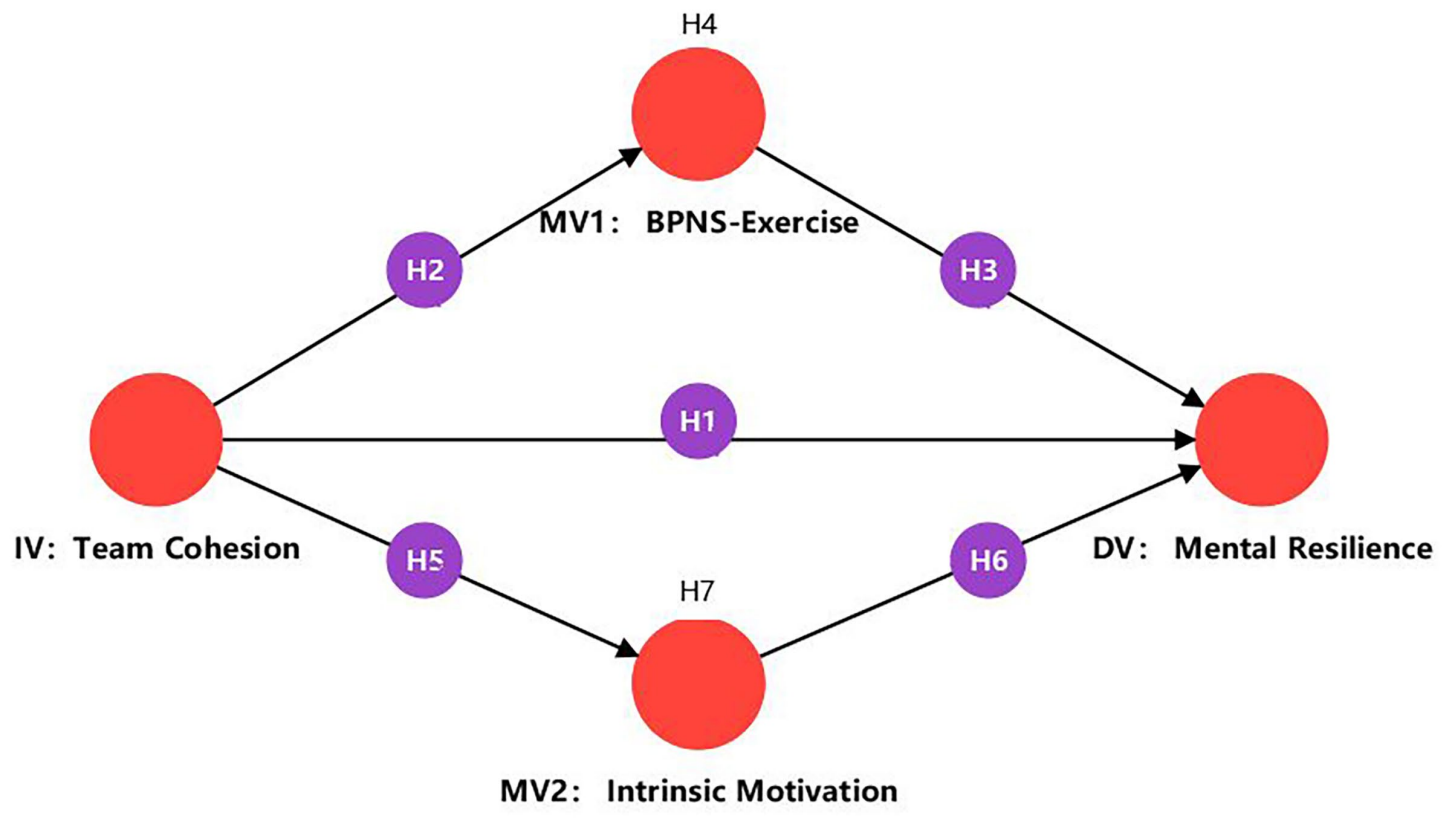
Theoretical model of the relationships between team cohesion, basic psychological need satisfaction, intrinsic motivation and mental resilience.

Figure 1 presents the conceptual model and the main hypothesised paths, which integrate Self-Determination Theory into a parallel mediation framework. In summary, the study tests seven hypotheses:

*H1*: Team cohesion is positively associated with vocational college students’ mental resilience and positively predicts mental resilience.

*H2*: Team cohesion positively predicts basic psychological need satisfaction in exercise.

*H3*: Basic psychological need satisfaction in exercise positively predicts mental resilience.

*H4*: Basic psychological need satisfaction in exercise mediates the relationship between team cohesion and mental resilience.

*H5*: Team cohesion positively predicts intrinsic motivation in ultimate frisbee courses.

*H6*: Intrinsic motivation positively predicts mental resilience.

*H7*: Intrinsic motivation mediates the relationship between team cohesion and mental resilience.

These hypotheses are tested in the subsequent Methods and Results sections using partial least squares structural equation modelling.

## 2 Methods

### 2.1 Study design and setting

This study adopted a cross-sectional survey design, and data were collected from ultimate frisbee elective PE classes in five vocational colleges in Guangxi, China. These colleges are typical employment-oriented institutions that offer a range of technical and professional programs, and ultimate frisbee is included as part of their regular physical education curriculum. All participants were full-time students who were officially enrolled in ultimate frisbee courses during the target semester.

The study focused on the classroom-based teaching context of ultimate frisbee, where students engage in regular practice, intra-class games and basic tactical exercises under the guidance of physical education instructors. Classes are commonly organised around small-group drills and game-like activities, during which students coordinate, communicate and cooperate to complete basic tactical tasks. All questionnaire items were anchored to this PE-class context, and students were instructed to respond based on their actual experiences in the ultimate frisbee course. The ultimate frisbee course(s) provide structured opportunities for team interaction and cooperation, which form the contextual basis for examining team cohesion, motivation-related variables and mental resilience in this research.

### 2.2 Participants and sampling

The target population of this study comprised college students enrolled in ultimate frisbee elective courses at five vocational colleges in Guangxi, China. Data were collected between September and November 2025, entirely within the Guangxi Zhuang Autonomous Region. The five selected institutions represent different professional domains and have all offered ultimate frisbee electives in their physical education programs over an extended period. These features ensure both the stability of course provision and, to some extent, the diversity and typicality of vocational colleges in Guangxi. According to the academic affairs systems of the participating institutions, the numbers of students registered for ultimate frisbee courses were 473, 314, 430, 507, and 465, respectively, yielding a total population of 2,189 students.

Given practical constraints in course scheduling and teaching management, we adopted convenience cluster sampling across five vocational colleges in Guangxi; therefore, the findings are most applicable to comparable vocational PE-class contexts, and broader generalization requires replication with multi-region samples. In each institution, the researchers consulted with the academic affairs office and course instructors to select several teaching classes that were offering ultimate frisbee electives in the current semester, and questionnaires were administered to all students within these classes who met the inclusion criteria.

Students were eligible to participate if they:

(1) were officially registered in an ultimate frisbee course in the current semester;
(2) were full-time students at one of the five vocational colleges; and.
(3) voluntarily agreed to take part and completed the entire questionnaire.

Exclusion criteria were:

(1) substantial missing data (more than 20% missing items in any of the scales);
(2) obviously abnormal response patterns or highly regular answers (e.g., selecting the same option for almost all items); and.
(3) duplicate questionnaires or responses that did not match the target population characteristics.

Assuming a 95% confidence level, an expected proportion of 0.50 and a margin of error of 0.05, the theoretical minimum sample size was approximately 384 participants (Rahman, 2023). To compensate for potential invalid or incomplete responses, we increased the planned sample size and administered the questionnaire online via QR-code access during ultimate frisbee course(s) across the five colleges (434 invitations/entries). In total, 420 online submissions were received; after applying the exclusion criteria, 390 valid questionnaires were retained for analysis. This sample size also exceeds the commonly recommended 10–20 observations per estimated path in PLS-SEM, ensuring adequate statistical power.

Participants’ demographic characteristics, including gender, grade level, age, college, and previous experience in team sports, are summarised in Table 1 (Demographic characteristics of participants) in the Results section.

**Table 1:** Demographic characteristics of participants (*N* = 390).

Variable	Category	*n*	%
Gender	Male	269	68.97
	Female	121	31.03
Grade	First year of college	215	55.13
	Second year of college	175	44.87
Age (year)	18	189	48.46
	19	180	46.15
	20	21	5.39
Institution	Institution A	84	21.54
	Institution B	56	14.36
	Institution C	77	19.74
	Institution D	90	23.08
	Institution E	83	21.28
Previous team sport experience (Yes/No)	Yes (1)	282	73.31
	No (2)	108	26.69

### 2.3 Measures

Four validated self-report instruments were used to assess team cohesion, basic psychological need satisfaction in exercise, intrinsic motivation, and mental resilience. Unless otherwise specified, higher scores indicate higher levels of the corresponding construct. All scales were administered in Chinese, using either previously validated Chinese versions or slightly adapted translations for the present context.

#### 2.3.1 Translation and pilot testing

All instruments were administered in Chinese. Where validated Chinese versions were available, they were used without modification. When only English originals were available, minor semantic adaptations were made so that the items better matched the ultimate frisbee course and vocational college context. Two researchers who were familiar with sport and psychology terminology independently produced initial Chinese translations. A bilingual researcher then back-translated the items into English. Any discrepancies between the original and back-translated versions were discussed in a small expert panel until agreement was reached, ensuring linguistic and cultural equivalence of the items.

Before the main data collection, a pilot test was conducted in an ultimate frisbee class at one vocational college with students similar to the target population. The pilot was used to check the clarity of instructions, wording of the items and response options, as well as the approximate time needed to complete the questionnaire. Based on students’ comments and item-level inspection, several minor wording adjustments were made to improve comprehensibility while preserving the original meaning of the items.

Preliminary psychometric analyses of the pilot data indicated that the four core scales performed well. For the latent variables of team cohesion (IV), basic psychological need satisfaction in exercise (MV1), intrinsic motivation (MV2) and mental resilience (MV3), Cronbach’s *α* values ranged from 0.846 (MV3) to 0.942 (IV). Composite reliability indices (rho_a and rho_c) were also high, with rho_a ranging from 0.868 to 0.944 and rho_c from 0.886 to 0.948. The average variance extracted (AVE) values exceeded or were close to the recommended threshold of 0.50 (IV = 0.503, MV1 = 0.580, MV2 = 0.700, MV3 = 0.566), suggesting satisfactory convergent validity of all four constructs. In addition, a preliminary PLS-SEM measurement model showed acceptable global fit (SRMR = 0.069; NFI = 0.694; d_ULS for the estimated vs. saturated model = 3.685 vs. 3.674; d_G = 1.885 for both), further supporting the adequacy of the measurement structure. Detailed reliability and validity indices based on the full sample are reported in the Results section.

#### 2.3.2 Team cohesion

Team cohesion was measured using the Chinese version of the Group Environment Questionnaire (GEQ) developed by Carron and colleagues (Brawley et al., 1987). The GEQ is a widely used instrument for assessing cohesion in sport teams and physical activity groups. In this study, we adopted the 18-item version suitable for school-based physical education settings. Items capture students’ perceptions of task-related and social aspects of cohesion, such as shared goals, willingness to remain with the team and quality of interpersonal relationships.

Each item was rated on a 9-point Likert scale ranging from 1 (“strongly disagree”) to 9 (“strongly agree”). Item scores were averaged (or summed) to obtain an overall team cohesion score, with higher scores reflecting stronger perceived cohesion in the ultimate frisbee class team. Previous research has supported the factor structure and reliability of the GEQ in Chinese sport and exercise contexts. In the present sample, the Chinese version of the GEQ showed excellent internal consistency (Cronbach’s *α* = 0.943).

#### 2.3.3 Basic psychological need satisfaction in exercise

Basic psychological need satisfaction in the exercise context was assessed using the Basic Psychological Needs in Exercise Scale (BPNS-Exercise), originally developed by Vlachopoulos and Michailidou (Cid et al., 2016). The scale measures the extent to which individuals feel that their three basic psychological needs—autonomy, competence, and relatedness—are satisfied during physical activity. Example items include statements such as feeling free to choose how to exercise (autonomy), feeling capable of successfully completing required tasks (competence), and feeling close to others while exercising (relatedness).

In this study, we used the 11-item version of the BPNS-Exercise. Participants responded to each item on a 5-point Likert scale ranging from 1 (“strongly disagree”) to 5 (“strongly agree”). Item scores were averaged to produce a total BPNS-Exercise score, with higher values indicating greater satisfaction of basic psychological needs in the context of the ultimate frisbee course. Previous validation studies have demonstrated good construct validity and reliability of the Chinese version of the BPNS-Exercise. In the present sample, the BPNS-Exercise scale demonstrated good internal consistency (Cronbach’s *α* = 0.903).

#### 2.3.4 Intrinsic motivation

Intrinsic motivation toward participation in the ultimate frisbee course was measured using the intrinsic motivation subscale of the Behavioural Regulation in Exercise Questionnaire-3 (BREQ-3) (de Oliveira Vanini et al., 2025). Within the Self-Determination Theory framework, this subscale captures the degree to which students engage in exercise for inherent interest, enjoyment and personal satisfaction rather than for external demands or rewards. Example items include statements such as “I participate in the frisbee class because I enjoy the exercise itself” and “I find frisbee practice interesting.”

Following the standard scoring procedure, we selected four items from the intrinsic motivation subscale. Students rated each item on a 7-point Likert scale ranging from 1 (“not at all true for me”) to 7 (“very true for me”). The intrinsic motivation score was computed as the mean of the four items, with higher scores reflecting higher levels of intrinsic motivation toward the frisbee course. The Chinese version of the BREQ-3 has shown satisfactory psychometric properties in previous research. In the present sample, the intrinsic motivation subscale of the BREQ-3 exhibited acceptable internal consistency (Cronbach’s *α* = 0.849).

#### 2.3.5 Mental resilience

Mental resilience was assessed using the Brief Resilience Scale (BRS) developed by Smith et al. (2008). The BRS focuses on individuals’ perceived ability to “bounce back” or recover from stress and adversity. It is suitable for non-clinical populations and has been widely used in student and health-related research (Smith et al., 2008).

The scale consists of six items, such as “I tend to bounce back quickly after hard times.” Participants rated each item on a 5-point Likert scale ranging from 1 (“strongly disagree”) to 5 (“strongly agree”). Items 2, 4 and 6 are negatively worded and were reverse-scored before analysis. The overall mental resilience score was obtained by averaging the six items, with higher scores indicating stronger resilience. The Chinese adaptation of the BRS has demonstrated good reliability and validity. In the present sample, the BRS showed acceptable internal consistency (Cronbach’s *α* = 0.820).

### 2.4 Procedure

Data collection was conducted by trained research assistants in ultimate frisbee course(s) at the five participating vocational colleges. At the beginning of each survey session, the research assistants briefly introduced the background and purpose of the study, explained the basic procedures for completing the questionnaire, and emphasised that participation was entirely voluntary, anonymous, and would not influence course grades.

Afterwards, the research team provided students with a written study information sheet and an electronic informed consent form. Students who had read the information and agreed to participate accessed the online questionnaire hosted on the Sojump (Wenjuanxing) platform by scanning a QR code with their mobile phones. The entire process was arranged during regular ultimate frisbee class time, and students completed the questionnaire independently.

During completion, course instructors did not participate in or intervene in the answering process, in order to reduce any perceived evaluation pressure. The research assistants moved around the classroom to monitor the process and, when necessary, clarified students’ questions about item comprehension, while avoiding any guidance that might influence their responses. Most students completed the questionnaire within approximately 5–10 min.

Once data collection was finished, the researchers immediately exported the raw data from the Sojump platform and conducted an initial screening. Questionnaires with serious missing data, clearly abnormal response patterns (e.g., selecting almost the same option for the entire questionnaire), or signs of duplicate submission were excluded. The cleaned dataset was then independently checked and cross-verified by two members of the research team to minimise potential errors in data processing, and the final validated dataset was used for statistical analysis.

This study was reviewed and approved by the Institutional Ethics Committee of UCSI University, Malaysia (Approval No.: UCSI-IEC-2025-FOSSLA-0055). All procedures were carried out in accordance with relevant institutional ethical guidelines and regulations. Electronic informed consent was obtained from all participants prior to questionnaire completion.

### 2.5 Data analysis

Data analysis was conducted in three stages: descriptive statistics, assessment of the measurement model, and evaluation of the structural model.

First, descriptive statistics for demographic variables and key study variables were calculated using IBM SPSS Statistics version 26.0. For the main continuous variables, we computed means, standard deviations, skewness and kurtosis to describe their distributions and to check for severe deviations from normality. The results are presented in Table 1 (sample characteristics) and Table 2 (descriptive statistics and correlations) in the Results section.

**Table 2:** Descriptive statistics and correlation matrix of core variables.

Variable	*M*	*SD*	Skewness	Kurtosis	1 (TC)	2 (BPNS)	3 (IM)	4 (MR)
1. TC	5.49	1.41	−0.19	−0.03	—	0.461**	0.661**	0.594**
2. BPNS	3.03	0.79	0.03	−0.55	0.461**	—	0.369**	0.470**
3. IM	4.25	1.49	−0.09	−0.81	0.661**	0.369**	—	0.586**
4. MR	3.04	0.98	0.13	−0.95	0.594**	0.470**	0.586**	—

*N* = 390. *M*, mean; SD, standard deviation. Skewness and kurtosis are based on unstandardized scores. Values above the diagonal represent Pearson correlation coefficients among the four variables. All correlations are significant at *p* < 0.01 (two-tailed). TC, team cohesion; BPNS, basic psychological need satisfaction in exercise; IM, intrinsic motivation; MR, mental resilience.

Second, partial least squares structural equation modelling (PLS-SEM) was performed with SmartPLS 4.0 to evaluate the reflective measurement models. We examined the outer loadings of all indicators and calculated Cronbach’s *α*, rho_A and composite reliability (ρ_c) to assess internal consistency. Convergent validity was evaluated using the average variance extracted (AVE), and multicollinearity was assessed using the variance inflation factor (VIF). Discriminant validity was examined by applying both the Fornell–Larcker criterion and the heterotrait–monotrait ratio of correlations (HTMT). The evaluation followed current PLS-SEM recommendations (e.g., reliability indices between 0.70 and 0.95, AVE ≥ 0.50, VIF < 3.3, HTMT < 0.85). The measurement model results are summarised in Table 3 (outer loadings and convergent validity) and Table 4 (discriminant validity indices).

**Table 3:** Factor loadings, average variance extracted (AVE), and reliability indices of the measurement model.

Factor	Outer loading	VIF	Cronbach’s alpha	rho_a	rho_c	AVE
Team_Cohesion_Q01 ≤ IV	0.756	2.141	0.943	0.945	0.949	0.511
Team_Cohesion_Q02 ≤ IV	0.656	1.671				
Team_Cohesion_Q03 ≤ IV	0.752	2.101				
Team_Cohesion_Q04 ≤ IV	0.620	1.537				
Team_Cohesion_Q05 ≤ IV	0.742	2.069				
Team_Cohesion_Q06 ≤ IV	0.679	1.780				
Team_Cohesion_Q07 ≤ IV	0.650	1.648				
Team_Cohesion_Q08 ≤ IV	0.779	2.355				
Team_Cohesion_Q09 ≤ IV	0.758	2.156				
Team_Cohesion_Q10 ≤ IV	0.670	1.701				
Team_Cohesion_Q11 ≤ IV	0.696	1.804				
Team_Cohesion_Q12 ≤ IV	0.760	2.211				
Team_Cohesion_Q13 ≤ IV	0.720	1.940				
Team_Cohesion_Q14 ≤ IV	0.729	1.996				
Team_Cohesion_Q15 ≤ IV	0.653	1.671				
Team_Cohesion_Q16 ≤ IV	0.733	2.000				
Team_Cohesion_Q17 ≤ IV	0.732	1.987				
Team_Cohesion_Q18 ≤ IV	0.758	2.146				
BPNES_Q01 ≤ MV1	0.732	2.521	0.903	0.907	0.919	0.509
BPNES_Q02 ≤ MV1	0.731	1.856				
BPNES_Q03 ≤ MV1	0.702	1.703				
BPNES_Q04 ≤ MV1	0.618	1.478				
BPNES_Q05 ≤ MV1	0.745	1.905				
BPNES_Q06 ≤ MV1	0.703	1.675				
BPNES_Q07 ≤ MV1	0.757	1.880				
BPNES_Q08 ≤ MV1	0.766	2.761				
BPNES_Q09 ≤ MV1	0.679	1.632				
BPNES_Q10 ≤ MV1	0.757	1.995				
BPNES_Q11 ≤ MV1	0.640	1.447				
Intrinsic_Motivation_Q01 ≤ MV2	0.835	1.873	0.849	0.851	0.898	0.689
Intrinsic_Motivation_Q02 ≤ MV2	0.835	1.926				
Intrinsic_Motivation_Q03 ≤ MV2	0.830	1.939				
Intrinsic_Motivation_Q04 ≤ MV2	0.819	1.871				
Mental_Resilience_Q01 ≤ DV	0.689	1.466	0.820	0.823	0.87	0.527
Mental_Resilience_Q02 ≤ DV	0.684	1.400				
Mental_Resilience_Q03 ≤ DV	0.744	1.592				
Mental_Resilience_Q04 ≤ DV	0.740	1.553				
Mental_Resilience_Q05 ≤ DV	0.736	1.550				
Mental_Resilience_Q06 ≤ DV	0.760	1.665				

**Table 4:** Discriminant validity of the latent variables based on Fornell–Larcker criterion and HTMT.

Factor	DV	IV	MV1	MV2
DV	0.726			
IV	0.597	0.715		
MV1	0.477	0.467	0.713	
MV2	0.59	0.663	0.375	0.830

Third, the structural model was evaluated in SmartPLS 4.0. We estimated the standardised path coefficients among latent variables and reported the coefficient of determination (*R*^2^) for each endogenous construct, as well as effect sizes (*f*^2^). To test the significance of direct and indirect effects, we used a bias-corrected bootstrap procedure with 5,000 resamples. This procedure provided standard errors, t-values and 95% bootstrap confidence intervals for each hypothesised path. Direct effects were considered statistically significant when the 95% confidence interval did not include zero (corresponding approximately to *p* < 0.05, two-tailed).

**Figure 2:**
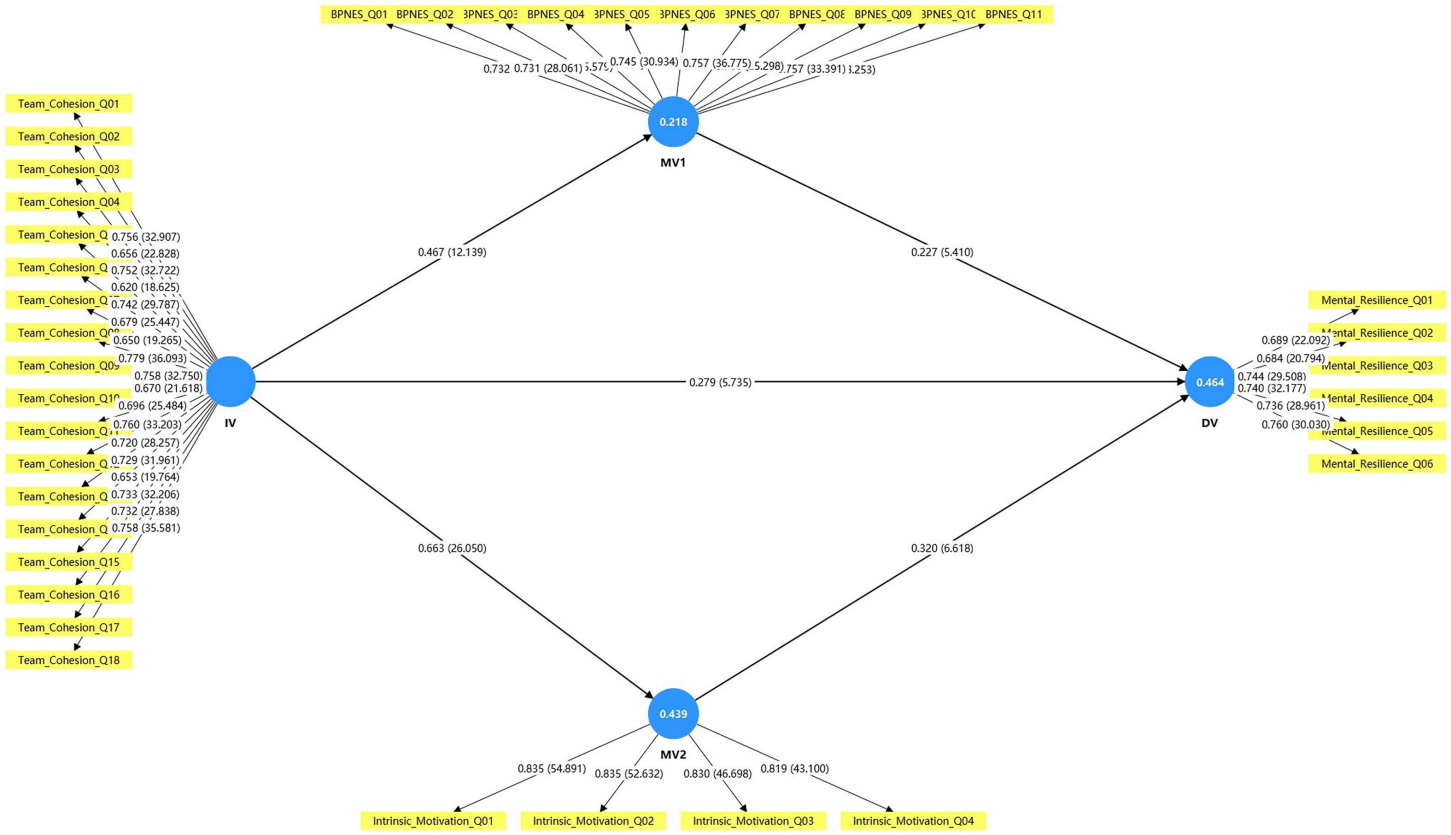
Structural model with standardized path coefficients and *R*^2^ values.

Parallel mediation effects of basic psychological need satisfaction in exercise and intrinsic motivation in the relationship between team cohesion and mental resilience were also examined within the same PLS-SEM framework. Indirect effects and total effects were computed and interpreted based on their bootstrap confidence intervals. The structural model results, including path coefficients and mediation estimates, are presented in Table 5 and Figure 2 in the Results section.

**Table 5:** Coefficients of determination (*R*^2^) for endogenous variables.

Endogenous variable	*R* ^2^	Adjusted *R*^2^
BPNS-Exercise	0.218	0.216
Intrinsic motivation	0.439	0.438
Mental resilience	0.464	0.459

## 3 Results

### 3.1 Sample characteristics

A total of 390 valid questionnaires were included in the final analysis (Table 1). Among the participants, 269 students were male (68.97%) and 121 were female (31.03%). With respect to grade level, 215 students (55.13%) were in their first year of college, and 175 students (44.87%) were in their second year. The majority of participants were 18 years old (*n* = 189, 48.46%) or 19 years old (*n* = 180, 46.15%), with a smaller proportion aged 20 years (*n* = 21, 5.39%). Participants were 18–20 years old, with a mean age of 18.57 ± 0.59 years.

Students were drawn from five vocational colleges in Guangxi, which are anonymised as Institutions A–E in Table 1. The proportions of students from each institution were 21.54% (*n* = 84) for Institution A, 14.36% (*n* = 56) for Institution B, 19.74% (*n* = 77) for Institution C, 23.08% (*n* = 90) for Institution D, and 21.28% (*n* = 83) for Institution E, respectively. Regarding previous experience in team sports, 282 students (73.31%) reported having participated in at least one team sport before taking the ultimate frisbee course, whereas 108 students (26.69%) indicated no prior team sport experience. Overall, the sample reflects a predominantly male cohort of first- and second-year vocational college students with a relatively high rate of prior exposure to team-based physical activities.

### 3.2 Descriptive statistics and correlations

#### 3.2.1 Descriptive statistics and normality

Table 2 presents the descriptive statistics for the four core variables, namely team cohesion (TC), basic psychological need satisfaction in exercise (BPNS-Exercise), intrinsic motivation (IM), and mental resilience (MR). Overall, the mean scores of all variables were above the midpoint of their respective scales, indicating that vocational college students enrolled in ultimate frisbee courses generally reported moderate to relatively high levels of perceived team cohesion, need satisfaction, intrinsic motivation, and mental resilience. The standard deviations were moderate. This suggests meaningful inter-individual variability without excessive dispersion.

**Figure 3:**
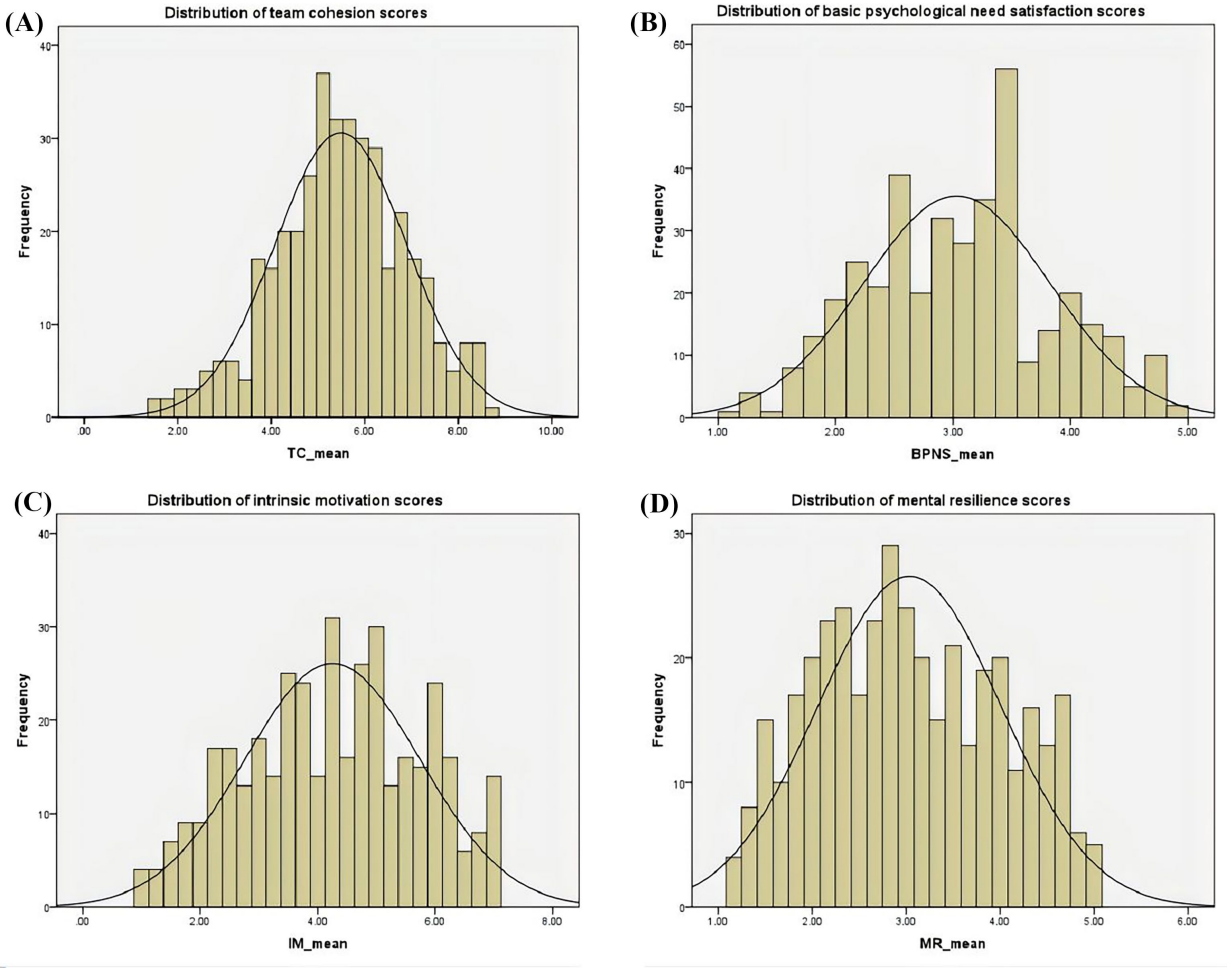
Distributions of team cohesion **(A)**, basic psychological need satisfaction in exercise **(B)**, intrinsic motivation **(C)**, and mental resilience **(D)**.

Regarding distributional properties, the skewness and kurtosis coefficients for all variables (see Table 2) fell within commonly accepted thresholds (absolute values largely below 2), indicating no severe skewness or extreme peakedness/flatness. Consistent with these statistics, the histograms with overlaid normal curves in Figure 3 show that team cohesion, basic psychological need satisfaction, intrinsic motivation, and mental resilience each display unimodal and approximately symmetric distributions, with only slight right-tail extensions at the higher score range. Taken together, these results support the assumption of approximate normality and provide an adequate basis for subsequent PLS-SEM estimation and mediation analyses.

#### 3.2.2 Correlations among main variables

As shown in Table 2, team cohesion (TC) was positively correlated with basic psychological need satisfaction in exercise (BPNS-Exercise), intrinsic motivation (IM) and mental resilience (MR), in line with the hypothesised directions. Students who perceived higher levels of team cohesion in the ultimate frisbee course(s) tended to report greater need satisfaction, stronger intrinsic motivation and higher mental resilience.

At the same time, BPNS-Exercise was positively associated with IM, and both were positively related to MR. This pattern suggests that students whose needs for autonomy, competence, and relatedness are more fully satisfied in the exercise context, and who participate in ultimate frisbee primarily out of interest and enjoyment, are more likely to exhibit stronger mental resilience when facing stress and challenges. Overall, the four core variables showed correlations of generally moderate to moderately high magnitude, indicating meaningful links among them while still maintaining adequate conceptual distinctiveness. This correlation pattern provides preliminary support for the hypothesised structural model in which team cohesion influences mental resilience through the parallel mediating effects of basic psychological need satisfaction in exercise and intrinsic motivation.

### 3.3 Measurement model

#### 3.3.1 Convergent validity and reliability

The reflective measurement model was first evaluated in terms of item loadings, convergent validity and internal consistency reliability. As shown in Table 3, the standardised outer loadings of all indicators on their respective latent variables ranged from 0.618 to 0.835. Most items exhibited loadings above the commonly recommended threshold of 0.70, and the few items with slightly lower loadings (but still above 0.60) were retained because they captured conceptually important aspects of the constructs and did not undermine the overall reliability indices.

With respect to convergent validity, the average variance extracted (AVE) values for the four latent constructs—team cohesion, basic psychological need satisfaction in exercise, intrinsic motivation and mental resilience—ranged from 0.509 to 0.689, all exceeding the cut-off value of 0.50. This indicates that each construct accounted for more than half of the variance in its indicators, supporting adequate convergent validity of the measurement model.

The reliability statistics also demonstrated satisfactory internal consistency. Cronbach’s *α* values for the four constructs ranged from 0.82 to 0.94, rho_A values were between 0.82 and 0.95, and composite reliability (ρ_c) values ranged from 0.87 to 0.95 (Table 3). All of these indices fell within the recommended interval of 0.70–0.95 for PLS-SEM applications, suggesting that the scales provided stable and internally consistent measurements of team cohesion, basic psychological need satisfaction, intrinsic motivation and mental resilience in this sample.

#### 3.3.2 Collinearity and discriminant validity

To examine potential multicollinearity among indicators, variance inflation factor (VIF) values were calculated in SmartPLS for all reflective items. As shown in Table 3, the indicator-level VIFs ranged from 1.40 to 2.76, which are well below the commonly used cut-off value of 3.3. These results suggest that there was no serious collinearity problem among the indicators. In addition, the full collinearity VIF diagnostics for the latent constructs did not indicate problematic common method bias according to established guidelines.

Discriminant validity was assessed using the Fornell–Larcker criterion together with the heterotrait–monotrait ratio of correlations (HTMT). As shown in Table 4, the square roots of the AVE values on the diagonal for mental resilience (DV = 0.726), team cohesion (IV = 0.715), basic psychological need satisfaction in exercise (MV1 = 0.713) and intrinsic motivation (MV2 = 0.830) are all larger than the corresponding inter-construct correlations in their rows and columns (ranging from 0.375 to 0.663). This pattern indicates that each latent variable shares more variance with its own indicators than with any other construct, thus meeting the Fornell–Larcker criterion. In addition, HTMT coefficients among the four constructs (not reported in the table) remained below the commonly recommended thresholds, providing further support for discriminant validity. Taken together, these findings suggest that multicollinearity is unlikely to be problematic and that the four core constructs are empirically distinct within the measurement model.

### 3.4 Structural model

#### 3.4.1 *R*^2^ and explanatory power

After establishing an adequate measurement model, the explanatory power of the structural model was examined. As shown in Table 5 and Figure 2, team cohesion and the two psychological mediators jointly accounted for a meaningful proportion of variance in the endogenous variables. Specifically, the model explained 21.8% of the variance in basic psychological need satisfaction in exercise (BPNS-Exercise; *R*^2^ = 0.218, adjusted *R*^2^ = 0.216), 43.9% of the variance in intrinsic motivation (IM; *R*^2^ = 0.439, adjusted *R*^2^ = 0.438), and 46.4% of the variance in mental resilience (MR; *R*^2^ = 0.464, adjusted *R*^2^ = 0.459). Overall, the *R*^2^ results suggest moderate explanatory power for mental resilience (*R*^2^ = 0.464) and intrinsic motivation (*R*^2^ = 0.439), and small-to-moderate explanatory power for BPNS-Exercise (*R*^2^ = 0.218), indicating that team cohesion and the two mediators account for a meaningful share of variance in this PE-class context while leaving room for other unmeasured determinants.

According to commonly used benchmarks for PLS-SEM, these values indicate that the model provides small-to-moderate explanatory power for BPNS-Exercise and moderate explanatory power for intrinsic motivation and mental resilience. In practical terms, the structural paths from team cohesion to the two mediators, and from team cohesion and the mediators to mental resilience, capture a substantial portion of the variability in students’ psychological functioning within the ultimate frisbee courses, while still leaving room for other unmeasured factors.

#### 3.4.2 Path coefficients

The structural path estimates are summarised in Table 6 and depicted in Figure 2. Team cohesion (TC) showed a positive and statistically significant direct effect on mental resilience (MR) (*β* = 0.279, *t* = 5.74, *p* < 0.001), supporting H1. TC also exerted significant positive effects on both basic psychological need satisfaction in exercise (BPNS-Exercise) and intrinsic motivation (IM) (*β* = 0.467, *t* = 12.14, *p* < 0.001; *β* = 0.663, *t* = 26.05, *p* < 0.001, respectively), thereby supporting H2 and H5. These findings indicate that students who perceived stronger team cohesion in the ultimate frisbee course(s) tended to report higher levels of need satisfaction and intrinsic motivation in the exercise context.

**Table 6:** Structural model assessment: Path coefficients, *t*-values, *p*-values, *f*^2^.

Factor	Original sample (O)	Mean	Std. dev	*t*	*p*	*f* ^2^
IV ≥ DV	0.279	0.281	0.049	5.735	0.00	0.073
IV ≥ MV1	0.467	0.471	0.038	12.139	0.00	0.279
IV ≥ MV2	0.663	0.665	0.025	26.05	0.00	0.783
MV1 ≥ DV	0.227	0.229	0.042	5.41	0.00	0.074
MV2 ≥ DV	0.32	0.319	0.048	6.618	0.00	0.106

In addition, both psychological mediators were positively related to MR. BPNS-Exercise had a significant positive path to MR (*β* = 0.227, *t* = 5.41, *p* < 0.001), and IM also showed a significant positive association with MR (*β* = 0.320, *t* = 6.62, *p* < 0.001), providing support for H3 and H6. Taken together, these results suggest that team cohesion not only has a direct link with students’ mental resilience, but is also associated with higher resilience through its positive relationships with need satisfaction and intrinsic motivation, a pattern that is further examined in the subsequent mediation analysis.

#### 3.4.3 Effect sizes and predictive relevance

Effect sizes for the structural paths were evaluated using Cohen’s *f*^2^ (Table 6). Team cohesion (IV) showed a small effect on mental resilience (DV; *f*^2^ = 0.073), a medium effect on basic psychological need satisfaction in exercise (MV1; *f*^2^ = 0.279), and a large effect on intrinsic motivation (MV2; *f*^2^ = 0.783). The paths from MV1 to DV and from MV2 to DV also yielded non-trivial effect sizes (*f*^2^ = 0.074 and *f*^2^ = 0.106, respectively), indicating that both basic psychological need satisfaction in exercise and intrinsic motivation make additional contributions to explaining students’ mental resilience beyond the direct effect of team cohesion.

To further assess out-of-sample predictive performance, the PLSpredict procedure was applied. As shown in Table 7, all endogenous constructs yielded positive *Q*^2^_predict values (0.209 for BPNS-Exercise, 0.436 for intrinsic motivation, and 0.350 for mental resilience), indicate non-trivial out-of-sample predictive relevance of the model in this course context. The RMSE and MAE values are of moderate magnitude, suggesting an acceptable level of prediction accuracy for the ultimate frisbee course(s).

**Table 7:** PLSpredict results for endogenous latent variables.

Factor	*Q*^2^ predict	RMSE	MAE
DV	0.35	0.809	0.656
MV1	0.209	0.893	0.713
MV2	0.436	0.755	0.614

DV, mental resilience; MV1, basic psychological need satisfaction in exercise; MV2, intrinsic motivation. *Q*^2^_predict > 0 indicates predictive relevance.

### 3.5 Mediation analysis

Parallel mediation analyses were conducted within the PLS-SEM framework to test whether basic psychological need satisfaction in exercise (BPNS-Exercise) and intrinsic motivation (IM) mediated the association between team cohesion (TC) and mental resilience (MR). Bias-corrected bootstrap procedures with 5,000 resamples were used to estimate indirect effects and their 95% confidence intervals (CIs).

As shown in Table 8, the indirect effect of TC on MR via BPNS-Exercise was positive and statistically significant (TC → BPNS-Exercise → MR: *β* = 0.106, 95% CI [0.064, 0.148]), indicating that higher perceived team cohesion was associated with greater satisfaction of basic psychological needs in the exercise context, which in turn predicted higher levels of mental resilience. This result supports the hypothesised mediating role of BPNS-Exercise.

**Table 8:** Indirect, direct, and total effects with 95% bootstrap confidence intervals.

Path	Original sample (O)	Mean	2.50%	97.50%
IV ≥ MV1 ≥ DV	0.106	0.108	0.064	0.148
IV ≥ MV2 ≥ DV	0.212	0.212	0.149	0.278

Similarly, the indirect effect of TC on MR through IM was also positive and significant (TC → IM → MR: *β* = 0.212, 95% CI [0.149, 0.278]), with the confidence interval again not crossing zero. This suggests that students who perceived stronger team cohesion tended to report higher intrinsic motivation toward the ultimate frisbee course, which was associated with enhanced mental resilience, thereby confirming the proposed mediating role of IM.

Importantly, after both mediators were included in the model, the direct path from TC to MR remained statistically significant (*β* = 0.279, *t* = 5.735, *p* < 0.001), indicating partial mediation rather than full mediation, that is, TC retains a direct association with MR beyond what is explained by the two mediators in this cross-sectional study, which suggests that TC may also influence MR through other unmeasured classroom social processes (e.g., peer support and a sense of belonging). Taken together, these findings support a partial parallel mediation model, in which TC influences MR both directly and indirectly through two concurrent psychological mechanisms: (i) promoting basic psychological need satisfaction in exercise and (ii) enhancing intrinsic motivation. In practical terms, a more cohesive team environment in ultimate frisbee course(s) appears to foster students’ psychological resilience partly by supporting their autonomy, competence and relatedness needs and by strengthening their intrinsic motivation, while also exerting an additional direct impact on resilience beyond these mediating processes.

## 4 Discussion

The present study, conducted in the context of ultimate frisbee courses at five vocational colleges in Guangxi, examined how team cohesion relates to college students’ mental resilience and tested the parallel mediating roles of basic psychological need satisfaction in exercise and intrinsic motivation. From a Self-Determination Theory perspective, team cohesion may promote mental resilience through two parallel processes—enhancing BPNS-Exercise and intrinsic motivation. In the results, the indirect effect via intrinsic motivation was stronger than that via BPNS-Exercise (*β* = 0.212 vs. 0.106), indicating that motivation was the more influential pathway in this PE-class context. Meanwhile, the direct path remained significant, suggesting that team cohesion may also operate through additional unmeasured social-support processes. Using PLS-SEM, we found that team cohesion had a significant direct effect on mental resilience, while also indirectly predicting resilience through both need satisfaction and intrinsic motivation. The model explained a moderate proportion of variance in the two mediators and in mental resilience, indicating that the proposed framework captures psychological processes operating in cooperative physical education settings.

Viewed in relation to previous research, the direct association between team cohesion and mental resilience observed in this study is broadly consistent with a growing body of evidence from team sport contexts. Prior studies have shown that athletes who perceive stronger cohesion within their teams tend to report better coping, lower stress and higher resilience, as cohesive teams provide emotional support, shared goals and a stronger sense of belonging (Meneghel et al., 2016). The results extend these patterns to vocational college students participating in a cooperative physical education course, rather than elite athletes or competitive sport teams. Compared with prior work that situates ultimate frisbee within university PE classes [e.g., (Xu, 2025)], the present study further brings in-class team cohesion into the explanatory chain and provides mechanism-oriented evidence linking a team process variable to mental resilience in a vocational PE context. In addition, earlier work has often examined either basic psychological need satisfaction or intrinsic motivation in isolation. By incorporating both constructs into a single parallel mediation model, the present study reveals that need satisfaction and intrinsic motivation operate simultaneously rather than competitively, thus offering a more nuanced picture of how social environments in physical education shape students’ psychological functioning.

From a theoretical perspective, the findings provide additional empirical support for the application of self-determination theory to physical education and mental health in vocational education settings. Self-determination theory proposes that social environments that support autonomy, competence and relatedness needs foster high-quality motivation and positive psychological outcomes (Ryan and Deci, 2000). In this study, team cohesion appears as a concrete social-contextual manifestation of such support: cohesive teams are more likely to provide opportunities for meaningful participation, shared decision making and mutual encouragement (Jones, 2024). This, in turn, promotes both basic need satisfaction and intrinsic motivation, which are associated with higher levels of mental resilience. The partial parallel mediation pattern observed in our model underscores that need satisfaction and intrinsic motivation represent distinct but interconnected psychological pathways linking team cohesion to resilience, thus enriching theoretical understanding of the “team cohesion – need satisfaction / intrinsic motivation – mental resilience” chain in cooperative sports courses.

Practically, the study offers several implications for physical education teachers and course administrators in vocational colleges. The results highlight the importance of intentionally cultivating a cohesive team climate in ultimate frisbee course(s), rather than focusing solely on technical skills or physical fitness. Teachers can adopt group-based tasks, small-sided games and rotational role assignments to encourage mutual support and shared responsibility, and can facilitate open communication to help students set collective goals and resolve conflicts constructively. At the same time, instructional strategies should be designed to support students’ autonomy (e.g., offering meaningful choices and explaining the rationale for tasks), competence (e.g., providing optimally challenging drills and formative feedback) and relatedness (e.g., fostering respectful, inclusive interactions) (Maddens et al., 2023). By creating an environment in which students feel connected, capable and self-determined, teachers can strengthen intrinsic motivation and, in turn, help students develop greater resilience when facing academic pressure, performance setbacks or interpersonal challenges (Buttigieg et al., 2025; Bülbül, 2025).

Despite its meaningful contributions, this study has several limitations that should be considered when interpreting the findings. First, the cross-sectional, self-report design does not allow for firm causal inferences about the direction of effects between team cohesion, need satisfaction, intrinsic motivation and mental resilience. It is possible, for example, that students with higher resilience are more likely to perceive the team as cohesive, rather than cohesion solely enhancing resilience. Second, the sample was drawn from five vocational colleges in a single province and focused on ultimate frisbee courses, which may limit the generalisability of the results to other regions, educational levels or types of physical activity. Third, although PLS-SEM is advantageous for handling complex models and modest deviations from normality, it simplifies some aspects of the error structure and may be complemented by other analytical approaches.

Building on these limitations, future research can extend the current work in several directions. In terms of design, longitudinal or intervention studies could be conducted to track changes in cohesion, psychological mediators and resilience over time, or to compare classes with deliberately enhanced team-building activities against regular instruction. Including multiple data sources, such as teacher ratings, behaviour indicators or peer assessments, would help reduce common method bias and provide a more comprehensive picture of students’ psychological functioning. Conceptually, it would be valuable to examine additional contextual factors—such as teaching styles, perceived teacher support, peer climate or school policies—and to explore potential moderating effects of gender, prior sport experience or academic major. Methodologically, multi-level modelling or covariance-based structural equation modelling could be used to cross-validate the present findings and to better capture class-level influences in cooperative physical education settings. Through such continued efforts, research can further clarify how cooperative sports like ultimate frisbee contribute to the promotion of mental resilience and broader mental health outcomes among vocational college students. This study treats team cohesion as a key social-process variable in PE classes and integrates it into an explanatory framework of mental resilience, extending prior work that has focused more on individual traits. The implication does not hinge on a single sport itself; rather, it points to a modifiable mechanism in team-based PE that can be shaped through instructional organization and peer interaction, which supports transfer to other settings.

## 5 Conclusion

This study, conducted in ultimate frisbee courses at five vocational colleges in Guangxi, examined how team cohesion is linked to college students’ mental resilience and tested the parallel mediating roles of basic psychological need satisfaction in exercise and intrinsic motivation. Using PLS-SEM with data from 390 students, we found that team cohesion was positively associated with mental resilience both directly and indirectly through the two psychological mediators. Students who perceived higher levels of cohesion in their frisbee teams tended to report greater satisfaction of autonomy, competence and relatedness needs in the exercise context, stronger intrinsic motivation toward the course and, in turn, higher levels of mental resilience (Sanchez-De Miguel et al., 2023). The final model provided moderate explanatory power for intrinsic motivation and mental resilience, and the significant indirect effects indicated a partial parallel mediation pattern. Overall, the findings suggest that in cooperative physical education settings, fostering a cohesive team climate and supporting students’ basic psychological needs and intrinsic motivation constitute important pathways for enhancing mental resilience among vocational college students.

More broadly, the results highlight that cooperative sports such as ultimate frisbee can play a meaningful role in promoting mental health, in addition to their traditional functions of improving physical fitness and sport skills (Xu, 2025). When physical education courses are designed to cultivate team cohesion and to systematically support students’ psychological needs, they may help students develop stronger adaptive resources for coping with academic pressure, performance setbacks and interpersonal challenges. For vocational colleges seeking to integrate mental health promotion into regular teaching activities, ultimate frisbee and similar cooperative activities offer a promising platform for embedding resilience-building elements into everyday physical education practice. These empirical findings provide evidence for the mental health–promoting potential of vocational physical education and lay a foundation for future intervention studies that systematically incorporate team-building, need-supportive teaching strategies and motivation-enhancing elements into cooperative sports programs.

## Data Availability

The raw data supporting the conclusions of this article will be made available by the authors, without undue reservation.
